# Reducing waste from single-use procedure packs in hospitals: A scoping review

**DOI:** 10.1177/0310057X251374693

**Published:** 2026-02-13

**Authors:** Alexandra R Seville, Katy JL Bell, Kristen M Pickles, Scott McAlister, Philomena Colagiuri, Luise Kazda, on behalf of the NSW Health Net Zero Clinical Leads Program

**Affiliations:** 1 NSW Health Net Zero, The Children’s Hospital at Westmead, Sydney, Australia; 2 NSW Ministry of Health Climate Risk & Net Zero, Sydney, Australia; 3 Wiser Healthcare Research Collaboration, Sydney School of Public Health, Faculty of Medicine and Health, The University of Sydney, Sydney, Australia; 4 The Healthcare Carbon Lab, Department of Critical Care, University of Melbourne, Melbourne, Australia; 5 Healthy Environments and Lives (HEAL) Global Research Centre, Health Research Institute, Faculty of Health, University of Canberra, Canberra, Australia

**Keywords:** Waste, single-use, scoping review, environmental impact, health system

## Abstract

Single-use items in pre-packaged procedure packs are disposed of after opening, regardless of whether they were used. We aimed to synthesise published evidence on evaluations of hospital-relevant interventions or comparisons targeting waste from procedure packs. We included intervention and aetiological observational studies, excluding purely descriptive studies, secondary studies and conference abstracts. We searched MEDLINE, Embase, CINAHL and Scopus from inception to 7 May 2024 and undertook forward and backward citation searches of included records. Of 1074 records retrieved, 14 studies met inclusion criteria. Eight were before–after quality improvement studies, five were comparative life cycle assessments and one was an audit. Studies reported on reductions in items in the pack (*n* = 8), change from single-use to reusable items (*n* = 3) and comparisons between different packs (*n* = 2) or individually packed items and packs (*n* = 1). Twelve studies reported on financial and waste impacts, six on environmental impacts and three on staff/patient impacts. Studies reported financial savings up to US$49.43 for reduced item packs and up to £7.22 for reusable packs versus single-use packs. Pack size and weight can often be much reduced, leading to less resource use and up to 2042g reduction in waste/pack and up to 860g less CO_2_ equivalent (CO_2_e) emissions. Switching instruments in packs from single-use to reusable reduced emissions by up to 90% where local energy was not predominantly from fossil fuels. No negative impacts on staff or patient outcomes were reported. Initiatives to reduce waste from single-use procedure packs may result in significant financial, resource use and CO_2_e emissions savings.

## Introduction

Hospitals are a major contributor to adverse environmental impacts with the healthcare system. Not only are they high consumers of energy and resources, but they also produce large amounts of physical waste.^1^ Plastics account for approximately one-third of hospital waste streams, and although 40–60% of this is potentially recyclable, most of it is currently sent to landfill as general or clinical waste.^2,3^ However, with recycling programs in place, at least half of the potentially recyclable waste could easily be recycled in operating rooms or intensive care units.^4,5^ For the New South Wales government alone, waste management for public health services is estimated to cost AU$16m per year as clinical waste, in particular, is more expensive to manage than recycling.^3^

Pre-packaged procedural packs, especially common in operating theatres, are found in all hospital areas wherever patient care involves a medical procedure. Historically, these packs comprised only reusable items, but the introduction of single-use materials (e.g. plastics, metals, cotton paper) has, anecdotally, seen an almost universal transition to single-use items in Australia. This transition has been justified on the grounds of infection control, frequent loss of reusable items within hospitals, and financial as well as labour savings often associated with single-use items that do not require reprocessing.^6,7^ There is evidence to suggest that often not all of the items in the pack are needed.^8–10^ However, once a procedure pack is opened, all items (whether used or not) are disposed of, creating substantial resource and financial waste.

Studies comparing single-use versus reusable instruments have found mixed results for resource waste, financial and environmental outcomes in Australia, depending on energy sources used for sterilisation processes (renewable energy versus fossil fuel based).^8,11^ In countries less dependent on fossil fuel derived energy, for example, New Zealand or France, where each kilowatt hour of energy produces 0.1 kg of CO_2_ emissions or less, moving from single use to reusable equipment would have much greater environmental impacts. However, reducing the number of items included in packs in the first place may partially avoid financial and environmental costs from manufacturing to disposal stages, and savings are irrespective of the type of energy source available. Thus, emphasising the practice of reducing, as part of the refuse, reduce, reuse, repurpose, recycle^8,12–16^ waste hierarchy,^8^ can notably decrease the environmental impact of healthcare.

Preliminary searches of MEDLINE, the Cochrane Database of Systematic Reviews and *JBI Evidence Synthesis* did not find any systematic or scoping reviews on reducing waste from single-use procedure packs. To help inform the development of our own intervention to reduce waste from central venous catheter (CVC) single-use packs in paediatric critical care, we set out to systematically search for, collate and synthesise existing evidence on interventions to reduce waste from single-use procedure packs in hospitals. We aimed to assess impacts on resource use and waste, financial costs, environmental outcomes (any impact on the environment, such as carbon emissions or water pollution) and staff or patient outcomes. Specifically, we sought to answer the question, what are the effects of interventions in hospitals to reduce waste from single-use procedure packs, on resource use, and financial, environmental and patient or staff impacts? We also collected information on potential barriers and facilitators to implementing these interventions.

## Methods

This scoping review was conducted in accordance with the Joanna Briggs methodology for scoping reviews^17^ and was reported in accordance with the Preferred Reporting Items for Systematic Reviews and Meta-Analyses Extension for Scoping Reviews (PRISMA-ScR) reporting guideline^18^ (Supplementary material Table S2 online). A review protocol was registered and published on Open Science Framework (https://doi.org/10.17605/OSF.IO/2MWKQ).

### Search strategy

An initial limited search of MEDLINE via Ovid was undertaken to identify key articles and key words and medical subject headings. With the support of an expert academic health librarian, a full search strategy was developed for MEDLINE and adapted for Embase, CINAHL and Scopus. We searched all four databases from inception to 7 May 2024, without restrictions on language (planning to utilise Google Translate for non-English manuscripts) (Supplementary material S3). We undertook backward and forward citation screening of included papers to locate any other potentially includable studies.

### Eligibility criteria

We outline the eligibility criteria according to the Participants, Concept, Context framing recommended for scoping reviews.^17^ The full eligibility criteria are shown in Supplementary material Table S1.

#### Participants

Studies with human participants of any age and sex and geographical location were eligible for inclusion with no focus on specific population groups.

#### Concept

The core concept was reducing waste from single-use procedure packs. Studies were ineligible if they did not report on procedure packs but, for example, only on individual single-use items such as personal protective equipment, plastic or metal surgery utensils, or pharmaceuticals. Studies examining packs made up exclusively of reusable items were also ineligible.

#### Context

Studies conducted either in a hospital setting or with specific relevance to a hospital setting were eligible. Community based healthcare settings such as primary care or specialist practices outside a hospital setting were ineligible. We considered studies on procedure packs from any clinical area, and from adult or paediatric care settings. There were no geographical restrictions.

#### Types of sources

We considered intervention, aetiological observational studies and comparative life-cycle assessments for inclusion. Purely descriptive observational studies (without attempt to estimate a causal effect), secondary studies and conference abstracts were all ineligible.

### Source of evidence screening and selection

All identified citations were collated and uploaded into Covidence and duplicates removed. Titles and abstracts were screened by one reviewer (PC or LK) against the predetermined eligibility criteria (Supplementary material Table S1). Potentially relevant sources were retrieved in full and two reviewers (LK and ARS) undertook independent assessment against the eligibility criteria. Reasons for exclusion at the full text stage were recorded. Any disagreements between the reviewers were resolved through discussion, with adjudication by a third reviewer (KMP) where necessary.

### Data extraction

Data was extracted by two reviewers (ARS and LK), one main extractor and one reviewer for each study, using a data extraction tool iteratively developed by the reviewers (Supplementary material Table S3) and piloted on three studies.^19–21^ We extracted data on the author, year, country, setting, study design, timing, participants/sample, study aim, comparator, intervention/exposure, outcomes and measurements, financial impact, resource use and waste, environmental impact, staff/patient impact, and any reported barriers or facilitators to implementation. Any uncertainties around data extraction were discussed and resolved by the extractors. A formal risk of bias assessment was not undertaken in this scoping review.

### Analysis and presentation of results

We tabulated characteristics of included studies and charted results, using the extraction template as a starting point. We used a narrative summary to describe the results according to five subthemes: financial impacts, resource use and waste impacts, environmental impacts, patient and staff impacts, and barriers and facilitators of intervention implementation.

## Results

**Figure 1.**
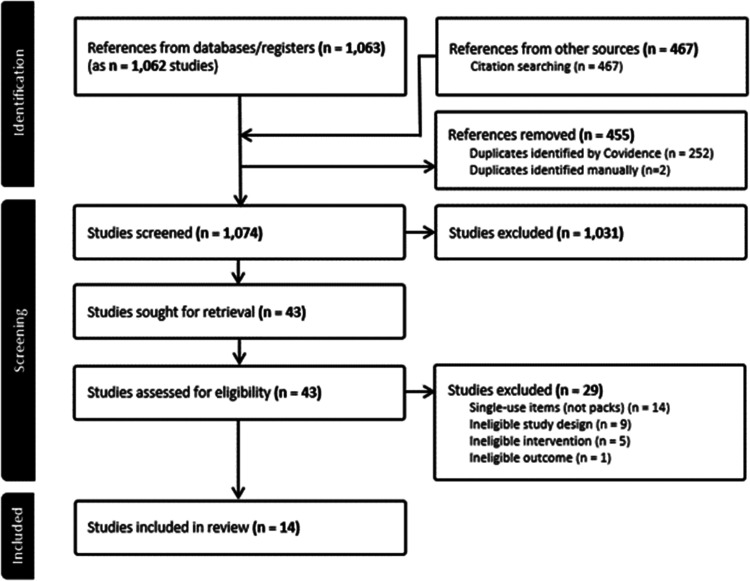
PRISMA flow chart detailing the review decision process, indicating the results from the searches, removal of duplicate citations, source selection, full retrieval and reasons for full text exclusions and final inclusions. PRISMA: Preferred Reporting Items for Systematic Reviews and Meta-Analyses.

The 1074 titles and abstracts retrieved from the database searches resulted in 43 records for full text screening. We excluded 29 of these (for reasons see Figure 1 and Supplementary material Table S4) and included 14 records in the review (Table 1^19–32^). Over half (*n* = 8) reported on quality improvement studies with a before and after design.^19,20,22–27^ The remaining studies were comparative life cycle assessments (*n* = 5)^21,28–31^ and one clinical audit (*n* = 1).^32^ One study included results applicable to intensive care units but did not describe an intervention.^31^ Two studies included results applicable to paediatric populations.^19,24^ All studies were published in English and from 2012 onwards: two each from 2012–2014,^24,31^ 2015–2017^22,28^ and 2018–2020^25,32^ and eight since 2021.^19–21,23,26,27,29,30^ Half of the studies were conducted in the USA (*n* = 7),^19,22,23,25,27,28,30^ with the remaining from the UK (*n* = 2),^20,24^ Sweden (*n* = 2),^29,32^ Austria (*n* = 1),^21^ Australia (*n* = 1)^31^ and France (*n* = 1).^26^ Most studies (*n* = 12) targeted single-use packs specific to a procedure performed within a specialty, three of which looked at hand surgeries^22,25,27^ and the others all looked at procedures in different specialties.^19–24,26,28,30,32^ The remaining two studies looked at single-use central line insertion packs.^29,31^ Eight studies evaluated a revised single-use procedure pack that included only frequently used items.^19,20,22–25,27,28^ To inform development of the revised packs, interviews,^22^ surveys,^20^ audits,^19,20,23^ item analysis^24,25,27^ and/or life cycle assessments^28^ were conducted to determine items for inclusion in the pack. Three studies investigated differences between single-use item packs and reusable-item packs,^29–31^ two studies made comparisons between existing packs,^21,32^ and one study evaluated different impacts between individually-packed single-use items compared with procedure packs containing the same items.^26^ Studies reported actual or potential impacts on financial costs, resource use and waste, environment, and patients and staff (Table 2^19–32^). Most studies reported on the potential for reducing financial costs (*n* = 12)^19,20,22–31^ and resource use and waste (*n* = 12).^19–28,31,32^ Some studies assessed environmental impacts (*n* = 6)^20,21,28–31^ or investigated patient or staff impacts (*n* = 4).^19,25,26,29^

**Table 1.** Study characteristics.

Author, year, country	Study period	Study design	Procedure(s)	Intervention	Comparator
Albert and Rothkopf, 2015, USA^22^	2012–2014	Quality improvement study (before/after)	Plastic surgeriesHand surgeries	Two revised, reduced-item, single-use packs based on interviews with surgeons	Two existing, single-use packs
Braschi et al, 2022, USA^23^	From January 2020 (unclear)	Quality improvement study (before/after)	Head and neck surgeriesLaparotomies	One revised, reduced-item, single-use pack based on unused supply audit	Two existing, single-use packs
Campion et al, 2015, USA^28^	Not reported	Comparative life cycle assessment	Vaginal births	One revised, reduced-item, single-use pack based on ‘Design for the Environment’ strategies	Fifteen existing, single-use packs
Cunningham et al, 2023, USA^19^	From March 2018 (unclear)	Quality improvement study (before/after)	Paediatric surgeries	Revised, reduced-item, single-use packs based on unused supply audit	Thirteen existing, single-use packs
Hemberg et al, 2023, Sweden^29^	Not reported	Comparative life cycle assessment	Central venous catheter insertion	None	One single-use only pack; one reusable only pack; one pack with reusable instruments and single-use textiles
Jabouri and Abbott, 2022, UK^30^	2019	Comparative life cycle assessment	Skin surgeries	None	One existing single-use; one existing reusable pack
Labib et al, 2024, UK^20^	Not reported	Quality improvement study (before/after)	Laparoscopic appendicectomies	One revised, reduced-item pack based on staff survey and audit	One existing mixed single-use/reusable pack
Lindskog and Männer, 2019, Sweden^32^	2013–2014	Audit	Hip replacement surgeries	None	Three existing, mixed single-use/reusable packs
McGain et al, 2012, Australia^31^	2011	Comparative life cycle assessment	Central venous catheter insertion	None	One existing, single-use pack vs one existing, reusable pack
Mouarbes et al, 2022, France^26^	Feb–Sep (2020 or 2021, unclear)	Quality improvement study (before/after)	ACL reconstruction	One new, single-use item pack (containing same instruments as before)	Single-packed, single-use instruments and surgical draping kit
Penn et al, 2012, USA^24^	July 2009–July 2010	Quality improvement study (before/after)	Paediatric (adeno-) tonsillectomies	One revised, reduced-item pack, single use based on item review and comparison with ambulatory centre	One existing, single-use pack
Thiel et al, 2019, USA^25^	May 2014–July 2015	Quality improvement study (before/after)	Hand surgeries	One revised, reduced-item, single-use pack based on item analysis; local anaesthesia only	Two existing, single-use packs; sedation and local anaesthesia
Velicki et al, 2023, USA^27^	2020–2021	Quality improvement study (before/after)	Hand surgeries	One revised, reduced-item, single-use pack based on item review	One existing, single-use pack
Winklmair et al, 2023, Austria^21^	2021	Comparative life cycle assessment	Cataract surgeries	None	Fifty-five existing, single-use packs from three suppliers

ACL: anterior cruciate ligament

**Table 2.** Impacts of Interventions to reduce waste from single-use procedure packs on financial costs, health resource use and waste, environmental outcomes, and on staff and patients, and reported barriers and facilitators for implementation.

StudyAuthor, year, country	Financial impacts	Resource use and waste	Environmental impacts	Staff and patient impacts	Barriers and facilitators
Albert and Rothkopf, 2015, USA^22^	Plastic pack cost/case: US$106.64 (pre) vs US$93.79 (post) (–12%); hand pack cost/case: US$49.36 (pre) vs US$44.56 (post) (–8%); estimated savings/year: US$41,844	Plastic pack items/case: 84 (pre) vs 43 (post) (–49%); hand pack items/case: 57 (pre) vs 23 (post) (–60%)	Not reported	Not reported	Not reported
Braschi et al, 2022, USA^23^	Pack cost/case: US$93.68/Laparotomy pack, US$89.51/head and neck pack (pre) vs US$46.88 (post) (–48% to –50%); estimated savings/year: US$45,719	Waste weight reduction/case: 1.088 kg; estimated waste weight reduction/year: 1105.4 kg	Not reported	Not reported	Barriers: concern regarding availability of instruments excluded from new pack
Campion et al, 2015, USA^28^	Pack costs: US$18.28 to US$26.47 (US packs, pre); revised pack: not costed but ‘will ultimately reduce the price of the custom pack, reduce the costs of disposal…’	Average pack weight: 1.226 kg (pre) vs 0.84 kg (post) (–31%)	Modelled average impact on eight categories as percent of average from US packs: 80% savings across all impact categories	Not reported	Not reported
Cunningham et al, 2023, USA^19^	Estimated savings/year: US$27,503 plus US$1,452 from waste disposal cost reductions	Estimated plastic waste weight reduction/year: 1829 kg	Not reported	No reports of excessive demand for missed items or prolonged operative time	Not reported
Hemberg et al, 2023, Sweden^29^	Cost/pack: €5.7 (RR^a^) (–38%), €9.1 (RS^b^) (–0.1%) vs €9.2 (SS^c^)Estimated savings/pack: €3.5 (RR^a^)	Not reported	Difference in resource use/pack: 65% (RR^a^), 20% (RS^b^) lower vs SS^c^ (in MJ primary^d^); difference in emissions/pack: 90% (RR^a^), 20% (RS^b^) lower vs SS^c^ (in kg CO_2_e); difference in ecosystem quality/pack: 85% (RR^a^), 70% (RS^b^) lower vs SS^c^ (in PDF*m^2^*years^e^)	Not reported	Not reported
Jabouri and Abbott, 2023, UK^30^	Cost/pack: £20.57 (single use) vs £13.35 (reusable) (–35%); estimated savings/year: £5253.55		Emissions/pack: 1.436 kg CO_2_e (single-use) vs 1.21 kg CO_2_e (reusable) (–16%); estimated savings/year: ∼6% (229.21 kg CO_2_e)	Not reported	Not reported
Labib et al, 2024, UK^20^	Cost/case: £99.31 (pre) vs £36.61 (post) (–63%); estimated savings/year: £31,350.32	Median number of single-use instruments used/case: 4 (pre) vs 1 item (post); single-use instrument usage/year: 2180 (pre) vs 705 items (post)	Emissions/case: 1.38 kg CO_2_e (pre) vs 0.52 kg CO_2_e (post) (–62%); estimated savings/year: 430 kg CO_2_e	Not reported	Barriers: belief that set had been updated recently, hesitance from colleagues on impact of updating the set, concerns regarding costs to purchase new instrumentsFacilitators: audit data demonstrating financial and environmental cost of current practice, involving colleagues in all decision-making regarding equipment purchase and set changes ensured buy-in and positive feedback
Lindskog and Männer, 2019, Sweden^32^	Not reported	Average weight of consumables/case: 5.7 kg (5.0–6.6 kg)Estimated average weight of consumables/case if using slimmest material: 3.9–4.5 kg (–10% to –41%)	Not reported	Not reported	Facilitators: discussing and finding solutions together with different professional groups, coordinating processes and learning from each otherBarriers: old routines, ignorance of better solutions
McGain et al, 2012, Australia^31^	Cost/pack: AU$6.35 (reusable) vs AU$8.65 (single-use) (–27%); estimated savings/pack: AU$2.30	Average pack weight: 0.627 kg (reusable) vs 0.171 kg (single-use) (+270%)	Emissions/pack: 407g CO_2_e (95% CI: 379 to 442g) (single-use) vs 1211g (95% CI: 1099 to 1323g) (reusable) (+198%); water use/pack: 2.5l (95% CI: 2.1 to 2.9l) (single-use) vs 27.7l (95% CI: 27.0 to 28.6l) (reusable) (+1008%)	Not reported	Not reported
Mouarbes et al, 2022, France^26^	Cost/surgery: €57.6 (pre) vs €105.9 (post) (+84%); estimated additional cost/year: €19,800	Number of packages/surgery: 36 (pre) vs 10 (post) (–72%); average weight of packaging/surgery: 0.221 kg (pre) vs 0.213 kg (post) (–4%)	Not reported	Preparation time of trolley/surgery: 4 min and 14 s (±40 s) (pre) vs: 2 s (±1 s) (post); opening time of instruments: 6 min and 31 s (±1 min 41 s) (pre) vs 18 s (±3 s) (post) Sorting of waste: 1 min 45 s (±18 s) (pre) vs 36 s (±5 s) (post) Number of movements to open instruments and sort waste: 186 movements (±13) (pre) vs 57 (±6) (post) Staff satisfaction rating: 9/10 (for intervention)	Not reported
Penn et al, 2012, USA^24^	Cost/pack: US$77.29 (pre) vs US$66.04 (post) (0.15%); estimated savings/year: US$17,149.50 plus US$830 savings in medical waste disposal costs	Average pack weight: 2.42 kg (pre) vs 1.70 kg (post) (–30%); estimated waste savings/year: 1034 kg	Not reported	Not reported	Barriers: unclear who sets policies for management of reusable instruments and evidence base of those policies, perception that packs reduce time and effort, preference by OR nurses
Thiel et al, 2019, USA^25^	Cost/pack: US$47.33 (pre) vs US$17.60 (post) (–63%); cost/case for all single-use items: US$104.69 (local only plus post pack) vs US$230.13 (sedation and local plus pre pack) (–55%)Estimated savings: US$29.73/pack, US$125.44/case	Average waste weight/case: 2.6 kg (2.4–2.8 kg (pre) vs 2.2 kg (post) (–15%); 2.2 kg (local only) vs 2.5 kg (sedation and local)	Not reported	Patient anxiety: local only (mean = 1.9, *n* = 38) vs sedation and local (mean = 1.2, *n* = 5, *P* = 0.0378) (scale 1–5); pain: local only (mean = 1.8;) vs sedation and local (mean =1.0, *P* < 0.005) (scale 1–5); satisfaction: local only (mean = 9.7, *n* = 40) vs sedation and local (mean = 9.3, *n* = 53, *P* 0.011) (scale 1–10)	Not reported
Velicki et al, 2023, USA^27^	Cost/pack: US$92.83 (pre) vs US$43.40 (post) to hospital (–53%); US$324.91 (pre) vs US$65.10 (post) to patients/insurance (–80%); estimated savings: US$49.43/pack (hospital); US$259.81/pack (patients)	Weight/pack: 4.938 kg (pre) vs 2.896 kg (post) (–41%); estimated waste savings/46 cases: 94 kg	Not reported	Not reported	Facilitator: based on positive experience of author, the only other hand surgeon routinely performing these procedures also started using the tailored local hand pack
Winklmair et al, 2023, Austria^21^	Not reported	Weight/pack: 0.5–1.17 kg; waste separation and disposal practices: approximately one-fifth of hospitals did not recycle for this surgery, one-half separated operating waste	Emissions/pack: 2.4 kg CO_2_e (with 100% incineration) vs 2.2 kg CO_2_e (with recycling); estimated emissions for 94% of packs sold in 2021 in Austria: 209,380 kg CO_2_e (with 100% incineration) or 195,804 kg CO_2_e (with recycling)	Not reported	Not reported

a RR = kit containing reusable instruments and textiles.

b RS = kit containing reusable instruments and single-use textiles.

c SS = kit containing single-use items.

d MJ primary = total amount of extracted non-renewable energy.

e PDF*m^2^*years = potentially disappeared fraction of species per square metre during a year.

CO_2_e: carbon dioxide equivalent; CI: confidence interval; OR: odds radio.

### Financial costs

Of the 12 studies reporting on financial impacts, 11 found potential or actual financial savings from the proposed or implemented interventions,^19,20,22–25,27–31^ while one study reported increased costs (intervention replaced individual items with a procedure pack).^26^ Seven quality improvement studies demonstrated cost savings after revising single-use packs to reduce the number of items^19,20,22–25,27^ Estimated annual savings ranged from US$17,980 (2012, tertiary children’s hospital, from packs used in 1854 tonsillectomies and adenotonsillectomies/year) to US$45,719 (2022, urban country hospital, from packs used in 1048 eligible general surgeries/year) in the USA^19,22–24^ and £31,350 in the UK (2023, an NHS (National Health Service) Trust, for packs used in 500 appendicectomies/year).^20^ One comparative life cycle assessment study of 15 different single-use packs for vaginal births reported costs per pack ranging from US$18.28 to US$26.47 (2015, packs from 12 US hospitals, two Thai hospitals and one non-profit medical supply organisation) but did not provide a cost for the revised, reduced-item pack.^28^ Three comparative life-cycle assessment studies comparing single-use with reusable item packs found potential financial savings with reusable items packs.^29–31^ One quality improvement study reported a cost increase of €48.30 per procedure and €19,800 per year (2022, orthopaedic department, large hospital, Toulouse, France) from moving from individually-packaged single-use items to a pre-packaged pack containing the same items for 410 anterior cruciate ligament (ACL) reconstruction/year.^26^

### Resource use and waste impacts

Twelve studies reported on potential or actual resource impacts from comparisons or interventions.^19–28,31,32^ A clinical audit study found differences of up to 1.800 kg in consumables per case (e.g. drapings, instruments, dressings) between three types of pack for hip replacements^32^ and a life cycle assessment study found differences of up to 0.670 kg in consumables between 55 types of pack for cataract surgeries,^21^ both signalling potential for reductions in resource use and waste generation. An Australian life cycle assessment of CVC packs reported that the mean weight for a reusable-item pack was 0.453 kg heavier than the single-use item pack, indicating potentially higher resource use.^31^ Eight studies compared resource impacts of existing, single-use item packs with those of newly created reduced-item single-use packs.^19,20,22–25,27,28^ One of these, a comparative life cycle assessment, found that the newly designed, reduced-item pack for vaginal births was 0.386 kg lighter than the mean weight of the existing 15 packs, thus creating less waste.^28^ The other seven, all quality improvement studies comparing single-use standard packs with reduced item ones, also reported reduced resource use. One UK study found that the number of single-use instruments used for 500 laparoscopic appendicectomies reduced from 2180 to 750 items per year.^20^ One US study found that revised packs for plastic and hand surgeries reduced items by nearly half for each plastic surgery (from 84 to 43) and hand surgery (57 to 23).^22^ Five studies that also reduced single-use items in packs reported waste weight reductions ranging from 0.400 kg^25^ to 2.042 kg^27^ per case (for hand,^25,27^ head and neck^23^ and adeno-tonsillectomy^24^ surgeries) and/or per year ranging from 1105 kg (USA, urban county hospital, 1048 head and neck surgeries)^23^ to 1829 kg (USA, medium-sized children’s hospital, 13 surgical packs revised).^19^ A French study found plastic packaging reduced non-significantly by 0.008 kg per pack when individually-packaged single-use items were replaced by a pre-packaged pack for ACL reconstructions.^26^

### Environmental impacts

Six studies measured environmental impacts,^20,21,28–31^ three of which reported only on carbon dioxide equivalent (CO_2_e) emissions.^20,21,30^ An Austrian life cycle assessment of cataract packs (2021) did not provide emissions estimates for the different packs used in Austria, but estimated that total CO_2_e emissions for all cataract packs purchased annually in the country could potentially be reduced by 3.25%, from 209,380 kg CO_2_e to 195,804 kg CO_2_e, if materials were recycled where possible.^21^ However, the authors estimated that if all cataract packs were to be reduced to the mean weight of the lower third of packages currently in use, CO_2_e emissions could be reduced by 34% (134,586 kg CO_2_e/year).^21^ One UK quality improvement study (2023) of reduced item single-use packs for laparoscopic appendicectomies reported carbon footprint savings of 0.860 kg CO_2_e emissions per case, and potential annual savings of 430 kg CO_2_e emissions for the NHS Trust (conducting 500 of these surgeries each year).^20^ A US life cycle assessment study (2015) found that a revised single-use procedure pack for vaginal births could result in 80% reduced environmental impact across eight categories (ozone depletion, global warming potential, smog, acidification, eutrophication, carcinogens, non-carcinogens, respiratory effects, ecotoxicity, cumulative energy demand) compared with the pre-existing, larger packs.^28^ A Swedish life cycle assessment study of CVC packs (2023) found that compared with single-use packs, reusable only and combined single-use and reusable packs could potentially reduce CO_2_e emissions by 90% and 20% and ecosystem quality (modelled in potentially disappeared fraction of species per square metre during a year) by 85% and 70%, respectively.^29^ In contrast, an Australian life cycle assessment study of CVC packs (2012) found that compared with single-use packs, reusable item packs increased CO_2_e emissions by 1.211 kg per pack, reflecting the high reliance on fossil fuel energy in Victoria, Australia at the time.^31^ This study also reported 11 times greater water requirements for reusable-item packs compared with single-use packs.^31^ Finally, one UK life cycle assessment study of skin surgery packs (2022) found that compared with single-use packs, reusable-item packs saved CO_2_e emissions by 0.226 kg per pack, with potential annual savings of 229.21 kg CO_2_e when conducting 62 procedures per week.^30^

### Staff and patient impacts

Three quality improvement studies reported impacts of interventions on staff and/or patients.^19,25,26,29^ A US study (2023) found that the introduction of a reduced item single-use paediatric surgery pack did not have any negative impact on operative debriefing reports; there was no indication for longer surgery times or complaints about missing items by staff using the refined packs.^19^ In another US quality improvement study (2019) of a revised single-use procedure pack for hand surgeries (in combination with wide-awake surgery) compared with a standard pack (in combination with traditional sedation and local anaesthetic), authors reported very similar patient anxiety and satisfaction scores.^25^ In the French quality improvement study (2022) investigating replacement of individually-packed single-use items with packs containing the same items, staff satisfaction with the revised pack was rated 9/10, with nurses’ movements and time spent on trolley preparation, opening instruments and waste sorting for each ACL surgery significantly decreased.

### Barriers/facilitators to interventions

Four studies reported on potential barriers to introducing interventions aimed at reducing waste from procedure packs.^20,23,24,27,32^ A US quality improvement study (2022) of revised procedure pack for head and neck surgeries and laparotomies reported some staff concerns around availability of instruments excluded from the new pack.^23^ A UK study of a revised item pack for laparoscopic appendectomies that reduced and changed the items (2023) reported some clinicians’ hesitancy due to beliefs that the set had been updated recently and did not need to be changed, and some were concerned about costs of purchasing new instruments.^20^ Another US quality improvement study of a revised pack used for adenotonsillectomies found ambiguities around policy responsibilities for management of reusable instruments and the evidence base of those policies, as well as staff perception of convenience of existing packs.^24^ A Swedish audit study of three existing mixed single-use and reusable packs for hip replacement surgeries (2019) mentioned the establishment of routines and unawareness of other solutions as potential barriers to change around procedure packs.^32^

Three studies mentioned potential facilitators to the process of implementing interventions on reducing waste from procedure packs.^20,27,32^ The UK study of a revised pack for laparoscopic appendectomies reported that collecting audit data to demonstrate the financial and environmental cost of current packs to colleagues, and involving staff in decision-making throughout the change process, ensured buy-in.^20^ Similarly, the audit study of existing packs for hip replacement surgeries reported that discussing and finding solutions together with different professional groups and coordinating processes in collaboration could facilitate change.^32^ Finally, the US quality improvement study of hand surgery packs reported that the positive experience of one of the authors with a reduced item pack persuaded other surgeons to switch to the refined pack as well.^27^

## Discussion

To our knowledge, this is the first published evidence review with a specific focus on reducing waste from single-use procedure packs. Of the 14 included studies, nearly all demonstrated substantial financial savings as a direct result of refining procedure packs to contain fewer items and/or more reusable components. Reported financial savings ranged from US$4.80 to US$49.43 per pack for reduced item packs and from AU$2.30 to £7.22 per pack for reusable packs versus single-use packs. Depending on the procedure, pack size and weight could often be much reduced, leading to less resource use: between 0.386 kg and 2.042 kg reduction in waste per pack and 0.226 kg to 0.860 kg less in CO_2_e emissions. Switching instruments from single use to reusable items in packs also reduced emissions by up to 90%, provided that local energy sources were not predominantly fossil fuels. No negative impacts on staff or patient outcomes were reported.

These findings align with reductions in CO_2_e emissions and financial costs found in studies investigating other (non-procedure pack) strategies to reduce use of single-use consumables and/or instruments for specific specialties.^13,14^ The reduce, reuse, recycle model – sometimes extended to also include refuse, repurpose or rethink – is often used as a framework for developing and ranking interventions to make healthcare more sustainable.^8,12–16^ Our results align with this model, showing that reducing oversupply of materials by minimising content in single-use procedure packs – such as removing unnecessary items, using smaller sized items (e.g. drapes) or replacing some with reusable items – seems to have the most significant impact. This approach not only eliminates waste but also reduces demand on procurement and, thus, manufacturing.^13,14^ Similar to findings from our study, reviews suggest that the ongoing success of sustainability interventions is strongly influenced by staff attitudes, levels of sustainability education and a ‘just in case’ culture, with clinicians expecting the availability of certain items present with no guarantee of use.^10,13,33–35^

Other studies comparing the environmental sustainability of single-use versus reusable items in clinical hospital settings produced varying results, largely dependent on the study country’s energy mix.^8,11^ Broadly, single-use items have been found to be less carbon intense than reusable items if the hospital’s main energy supply comes from fossil fuel.^8^ The importance of monitoring emissions across the entire healthcare system, rather than focusing on quality improvement in silos, has also been highlighted previously.^9,36^ However, to date, most of the available literature is specialty-based with interventions not extending across specialty, facility, state or national levels.^37^ Nevertheless, there is now sufficient de-carbonisation knowledge amongst specialties for initiatives to be translated across disciplines, where there is a lack of specialty-specific studies.^37^ The minimal allocation of funding for healthcare decarbonisation projects has previously been noted as a barrier to developing well designed, evidence-based, long-term initiatives to support clinicians and hospital managers in reducing carbon emissions from healthcare.^9^

Strengths of this review are the systematic approach used, based on best practice methods for scoping reviews including JBI and PRISMA methodologies, as well as a diverse multidisciplinary project team led by a clinician with expert content knowledge and supported by a health librarian. Our review also has some limitations. A single reviewer conducted all title and abstract screening, and we did not assess the risk of publication bias nor undertake risk of bias assessments of included studies. Studies that did not find positive impacts may be less likely to be published. Most included studies had weak study designs, predominantly quality improvement studies using before and after comparisons. Additionally, all studies were specialty-specific and carried out in high-income countries, which may limit the generalisability of results to other settings.

Our findings suggest that clinician-led initiatives to reduce over-consumption of single-use plastics can drive efficiencies in the health services they deliver and have tangible impacts on healthcare sustainability. Relatively simple changes to hospital or health district procurement policies and procedures could yield substantial benefits. Adopting a triple-bottom-line approach – considering profit, people and the planet – into procurement processes could ensure financial and environmental savings whilst benefitting staff and patients.^38,39^ Many of the included interventions were designed directly by clinical staff who had observed in their routine practice that pack contents were excessive and might be easily reducible without impacting negatively on clinical care. Our analysis of barriers and facilitators highlights the importance of ensuring buy-in from staff when designing packs as well as conducting regular periodical reviews of all procedure packs within healthcare facilities. Regular reviews of packs for suitability and avoidance of excess items and materials could be easily introduced as a standard quality assurance process to many facilities. Future research may benefit from improved design and reporting of interventions with theoretical foundations and, ideally, co-designed with clinicians and patients. A greater focus on patient and staff-relevant outcomes, as well as barriers and facilitators to change, is needed to support successful implementation and long-term change. Accurate and reliable outcome measures with environmental impacts measured by process-based life cycle assessment are also required.

## Conclusions

Interventions to reduce waste from single-use procedure packs might result in significant financial, resource use and CO_2_e savings.

## Supplemental Material

sj-pdf-1-aic-10.1177_0310057X251374693 - Supplemental material for Reducing waste from single-use procedure packs in hospitals: A scoping review Supplemental material, sj-pdf-1-aic-10.1177_0310057X251374693 for Reducing waste from single-use procedure packs in hospitals: A scoping review by Alexandra R Seville, Katy JL Bell, Kristen M Pickles, Scott McAlister, Philomena Colagiuri and Luise Kazda and on behalf of the NSW Health Net Zero Clinical Leads Program in Anaesthesia and Intensive Care
