# Supplementary material for: Reducing waste from single-use procedure packs in hospitals: A scoping review
Source: Anaesth Intensive Care. 2026 Feb 13;54(2):113–24. doi: 10.1177/0310057X251374693 (PMC12967391; doi:10.1177/0310057X251374693)
Supplement: sj-pdf-1-aic-10.1177_0310057X251374693 - Supplemental material for Reducing waste from single-use procedure packs in hospitals: A scoping review [file sj-pdf-1-aic-10.1177_0310057X251374693.pdf]

REDUCING WASTE FROM SINGLE-USE PROCEDURE PACKS IN HOSPITALS: A SCOPING – SUPPLEMENTS

1. **Table S1. Inclusion and Exclusion Criteria**
2. **Table S2. PRISMA-ScR Checklist**
3. **S3. Search Strategies**
4. **Table S3. Data extraction instrument**
5. **Table S4. Sources excluded following full-text review**

Table S1: Inclusion and Exclusion Criteria

Aspect	Inclusion criteria	Exclusion criteria
Population	• Human participants, any age, sex	• Non-human subjects
Intervention/ exposure	• Reducing waste from single-use packs	• No intervention or exposure (e.g. just a baseline measure but no change is recorded)<br>• Waste from other clinical procedures/ products (e.g. pharmaceutical, PPE)<br>• Waste from individual single-use items that are not part of a pack<br>• Designed purely to reclassify waste, not reduce it
Comparator	• Any comparator or no comparator	• N/A
Outcome	• Reduction in waste, financial, environmental impact<br>• Implementation outcomes (e.g. patient outcomes, acceptability, feasibility, engagement)	• Other outcomes from targeting single-use packs (e.g. only financial costs)
Setting	• Results are relevant to any hospital setting<br>• Any country	• Results are not relevant to a hospital setting
Study design	• RCTs<br>• Non-randomised intervention studies<br>• Observational studies attempting to estimate causal effect (comparative studies)	• Reviews<br>• Not peer reviewed<br>• Descriptive observational study - no attempt to estimate a causal effect

Table S2: PRISMA-ScR Checklist

SECTION	ITEM	PRISMA-ScR CHECKLIST ITEM	REPORTED ON PAGE #
**TITLE**			
Title	1	Identify the report as a scoping review.	1
**ABSTRACT**			
Structured summary	2	Provide a structured summary that includes (as applicable): background, objectives, eligibility criteria, sources of evidence, charting methods, results, and conclusions that relate to the review questions and objectives.	2
**INTRODUCTION**			
Rationale	3	Describe the rationale for the review in the context of what is already known. Explain why the review questions/objectives lend themselves to a scoping review approach.	4
Objectives	4	Provide an explicit statement of the questions and objectives being addressed with reference to their key elements (e.g., population or participants, concepts, and context) or other relevant key elements used to conceptualize the review questions and/or objectives.	4
**METHODS**			
Protocol and registration	5	Indicate whether a review protocol exists; state if and where it can be accessed (e.g., a Web address); and if available, provide registration information, including the registration number.	4
Eligibility criteria	6	Specify characteristics of the sources of evidence used as eligibility criteria (e.g., years considered, language, and publication status), and provide a rationale.	4/5
Information sources*	7	Describe all information sources in the search (e.g., databases with dates of coverage and contact with authors to identify additional sources), as well as the date the most recent search was executed.	4
Search	8	Present the full electronic search strategy for at least 1 database, including any limits used, such that it could be repeated.	S3
Selection of sources of evidence†	9	State the process for selecting sources of evidence (i.e., screening and eligibility) included in the scoping review.	5
Data charting process‡	10	Describe the methods of charting data from the included sources of evidence (e.g., calibrated forms or forms that have been tested by the team before their use, and whether data charting was done independently or in duplicate) and any processes for obtaining and confirming data from investigators.	6
Data items	11	List and define all variables for which data were sought and any assumptions and simplifications made.	6 & Table S3
Critical appraisal of individual sources of evidence§	12	If done, provide a rationale for conducting a critical appraisal of included sources of evidence; describe the methods used and how this information was used in any data synthesis (if appropriate).	N/A
Synthesis of results	13	Describe the methods of handling and summarizing the data that were charted.	6

SECTION	ITEM	PRISMA-ScR CHECKLIST ITEM	REPORTED ON PAGE #
**RESULTS**			
Selection of sources of evidence	14	Give numbers of sources of evidence screened, assessed for eligibility, and included in the review, with reasons for exclusions at each stage, ideally using a flow diagram.	6 & Figure 1
Characteristics of sources of evidence	15	For each source of evidence, present characteristics for which data were charted and provide the citations.	6 & Table 1
Critical appraisal within sources of evidence	16	If done, present data on critical appraisal of included sources of evidence (see item 12).	N/A
Results of individual sources of evidence	17	For each included source of evidence, present the relevant data that were charted that relate to the review questions and objectives.	Table 2
Synthesis of results	18	Summarize and/or present the charting results as they relate to the review questions and objectives.	7-11
**DISCUSSION**			
Summary of evidence	19	Summarize the main results (including an overview of concepts, themes, and types of evidence available), link to the review questions and objectives, and consider the relevance to key groups.	11-13
Limitations	20	Discuss the limitations of the scoping review process.	12/13
Conclusions	21	Provide a general interpretation of the results with respect to the review questions and objectives, as well as potential implications and/or next steps.	13
**FUNDING**			
Funding	22	Describe sources of funding for the included sources of evidence, as well as sources of funding for the scoping review. Describe the role of the funders of the scoping review.	13/14

From: Tricco AC, Lillie E, Zarin W, O'Brien KK, Colquhoun H, Levac D, et al. PRISMA Extension for Scoping Reviews (PRISMAScR): Checklist and Explanation. Ann Intern Med. 2018;169:467–473. doi: 10.7326/M18-0850.

S3: Search Strategies

Scopus - run on 9/05/2023, updated on 07/05/2024
"intensive care" OR "critical care" OR icu OR emergency OR "casualty depart*" OR "accident depart*" OR hospital* AND (("single use" OR "non-reusable" OR disposable) W/5 (kit OR kits OR equip* OR pack* OR catheter* OR supplies)) OR "single line insertion kit*" AND (reduc* W/3 waste) OR environment* OR sustainab* OR "net zero" OR footprint

MEDLINE via Ovid, Embase via Ovid, CINAHL via Ebsco – all run on 9/5/2023 updated on 07/05/2024

Table S3: Data extraction instrument

Author, year, country	Setting	Study design	Timing	Participants/ Sample	Aim	Comparator	Intervention/ Exposure	Outcomes & measurements	Resource/ Financial impact	Environmental impact	Staff/ Patient impact	Barriers/ Facilitators

Table S4. Sources excluded following full-text review

Authors	Year	Title	Journal	Exclusion reason
Becker, C.	2002	Going to waste. One surgeon's trash can be a hospital's treasure as providers figure out how to minimize opening supplies that don't get used	Modern healthcare	Ineligible study design
Bhogal, S.; Lalonde, D.; Baratz, M.	2022	Waste Mitigation: A Marriage of Cost Savings and Sustainability	Operative Techniques in Orthopaedics	Ineligible study design
Boberg, L.; Singh, J.; Montgomery, A.; Bentzer, P.	2022	Environmental impact of single-use, reusable, and mixed trocar systems used for laparoscopic cholecystectomies	PLoS ONE	Waste from single-use items (not packs)
Byrne, C.; Pley, C.; Schorscher, P.; Brandon, Z.; Gatumbu, P.; Mallinson, C.; Vaghela, M.	2023	A mixed-methods analysis of the climate impact, acceptability, feasibility and cost of switching from single-use pulp to reusable plastic trays in a large NHS trust.	Future healthcare journal	Waste from single-use items (not packs)
Chin, CJ; Sowerby, LJ; John-Baptiste, A; Rotenberg, BW	2014	Reducing otolaryngology surgical inefficiency via assessment of tray redundancy	Journal of otolaryngology - head & neck surgery	Waste from single-use items (not packs)
Crosby, L.; Lortie, E.; Rotenberg, BW.; Sowerby, LJ.	2019	Surgical Instrument Optimization to Reduce Instrument Processing and Operating Room Setup Time.	Otolaryngology-head and neck surgery	Waste from single-use items (not packs)
de Ridder, EF.; Friedericy, HJ.; van der Eijk, AC.; Dankelman, J.; Jansen, FW.	2022	A New Method to Improve the Environmental Sustainability of the Operating Room: Healthcare Sustainability Mode and Effect Analysis (HSMEA)	Sustainability	Ineligible study design
DiConsiglio, J.	2008	Reprocessing SUDs reduces waste, costs	Materials management in health care	Ineligible study design
Engler, ID.; Koback, FL.; Curley, AJ.	2023	Value-Based, Environmentally Sustainable Anterior Cruciate Ligament Surgery	Clinics in Sports Medicine	Ineligible study design
English, N.	1996	Reprocessing disposables: one strategy to balance cost reduction and quality patient care	Today's surgical nurse	Ineligible study design
Farrokhi, F.R.; Gunther, M.; Williams, B.; Blackmore, C.C.	2015	Application of lean methodology for improved quality and efficiency in operating room	Journal for Healthcare Quality	Waste from single-use items (not packs)

		instrument availability		
Fogliatto, FS; Anzanello, MJ; Tonetto, LM.; dos Santos Schneider, DS; de Magalhaes, AMM.	2019	Lean-healthcare approach to reduce costs in a sterilization plant based on surgical tray rationalization	Production Planning & Control	Ineligible intervention;
Friedericy, HJ.; van Egmond, CW.; Vogtlander, JG.; van der Eijk, AC.; Jansen, FW.	2021	Reducing the Environmental Impact of Sterilization Packaging for Surgical Instruments in the Operating Room: A Comparative Life Cycle Assessment of Disposable versus Reusable Systems	Sustainability	Waste from single-use items (not packs)
Harvey, L. F. B.; Smith, K. A.; Curlin, H.	2017	Physician Engagement in Improving Operative Supply Chain Efficiency Through Review of Surgeon Preference Cards	Journal of Minimally Invasive Gynecology	Waste from single-use items (not packs)
Kodumui, P; Jesudason, P; Houghton, J	2022	SusQI Project Report: The Upper 'Hand' of Sustainability: Reducing the Carbon Footprint in Hand Surgery	Green Surgery Challenge	Waste from single-use items (not packs)
Kyle, E.	2024	Sustainability in the Perioperative Practice Setting.	AORN journal	Ineligible study design
Leiden, A; Cerdas, .; Noriega, D.; Beyerlein, .; Herrmann, C	2020	Life cycle assessment of a disposable and a reusable surgery instrument set for spinal fusion surgeries	Resources, Conservation and Recycling	Ineligible intervention
Lodi, C. A.; Malagoli, M.; Gilioli, M.; Bandini, R.; et al	2020	The environmental impact of disposables in a new configuration of hemodialysis (HD) system	Nephrology Dialysis Transplantation	Waste from single-use items (not packs)
McGain, F.; Story, D.; Lim, T.; McAlister, S.	2017	Financial and environmental costs of reusable and single-use anaesthetic equipment	British Journal of Anaesthesia	Waste from single-use items (not packs)
Meiklejohn, D. A.; Chavarri, V. M.	2021	Cold Technique in Adult Tonsillectomy Reduces Waste and Cost	Ear, Nose and Throat Journal	Waste from single-use items (not packs)
Parker, EB.; Bluman, EM.; Chiodo, CP.; Martin, EA.; Smith, JT.	2024	Carbon Footprint of Minor Foot and Ankle Surgery: A Randomized Controlled Trial.	Foot & ankle orthopaedics	Ineligible intervention

Pesigan, Precy; Chen, Henry; Bajaj, Arjun A.; Gill, Harcharan S.	2021	Cost Savings in Urology Operating Rooms by Editing Surgeon Preference Cards	Quality Management in Health Care	Waste from single-use items (not packs)
Rizan, C.; Bhutta, M.F.	2022	Environmental impact and life cycle financial cost of hybrid (reusable/single-use) instruments versus single-use equivalents in laparoscopic cholecystectomy	Surgical Endoscopy	Waste from single-use items (not packs)
Sikka, R. S.; Fischer, D. A.; Swiontkowski, M. F.	2005	Reprocessing single-use devices: An orthopaedic perspective	Journal of Bone and Joint Surgery	Ineligible study design
Szirt, R.; Monjur, MR.; McGovern, L.; Charlesworth, K.; O'Connor, S.; Weaver, JC.; Coughlan, JJ.	2022	Environmental Sustainability in the Cardiac Catheter Laboratory.	Heart, lung & circulation	Ineligible study design
Thiel, CL.; Woods, NC.; Bilec, MM.	2018	Strategies to Reduce Greenhouse Gas Emissions from Laparoscopic Surgery.	American journal of public health	Ineligible intervention
Van Demark, R. E.; Smith, V. J. S.; Fiegen, A.	2018	Lean and Green Hand Surgery	Journal of Hand Surgery	Ineligible intervention
Walchak, A. C.; Porembski, M. A.; Lansinger, Y. C.; Ruffin, R. A.; et al	2021	Operating room supply waste in elective hand surgery	Perioperative Care and Operating Room Management	Ineligible outcomes
Wolstencroft, PW; Zacher, NC; Scotellaro, K; Centkowski, S; Kwong, BY	2023	Development of a Framework for Addressing Skin Biopsy Tray Waste in Dermatology Clinics: A Quality Improvement Study.	JAMA dermatology	Waste from single-use items (not packs)
